# Repeat Biopsy to Assess Duodenal Healing in Children With Celiac Disease and Eosinophilic Gastrointestinal Disorders

**DOI:** 10.1097/PG9.0000000000000097

**Published:** 2021-07-12

**Authors:** Kaitlin Payne, Lydia Ramharack, Patricia Bierly, Kara Feigenbaum, Janel Steinhoff, Karen Hlywiak, Ann Farrara, Ritu Verma, Arunjot Singh, Lisa M. Fahey

**Affiliations:** From the * Division of Gastroenterology, Hepatology, and Nutrition, The Children’s Hospital of Philadelphia, Philadelphia, Pennsylvania; † Sidney Kimmel Medical College at Thomas Jefferson University, Philadelphia, Pennsylvania; ‡ University of Chicago, Pediatric Gastroenterology, Hepatology, and Nutrition, Chicago, Illinois; § Perelman School of Medicine, University of Pennsylvania, Philadelphia, Pennsylvania.

**Keywords:** pediatrics, tTG-IgA, endoscopy, villous blunting, eosinophilic esophagitis

## Abstract

The aim of the study was to determine the correlation between duodenal mucosal biopsies and tissue transglutaminase immunoglobulin A (tTG-IgA) levels in pediatric patients with biopsy-confirmed celiac disease (CD) and eosinophilic gastrointestinal disorders (EGID) who have had repeat duodenal biopsies after initiating a gluten-free diet.

**Methods:**

A retrospective chart review was performed of children with CD and EGID seen at the Children’s Hospital of Philadelphia between 2003 and 2018. Data collected included duodenal biopsy pathology, celiac serology including tTG-IgA, and symptom reports. Duodenal healing was defined as normal villous architecture and no intraepithelial lymphocytes. These data were compared with tTG-IgA level. Data were analyzed with Fisher exact test and *t* test methods.

**Results:**

Thirty-nine patients had normal IgA and diagnoses of both CD and EGID. At second biopsy, 44% (17/39) of patients showed no histologic evidence of active CD and 36% (14/39) of patients had negative tTG-IgA values. Sixty percent (9/15) of patients with no evidence of CD on biopsy had abnormal tTG-IgA levels, and 57% (8/14) of patients with normal tTG-IgA levels had evidence of active disease on biopsy.

**Conclusions:**

The data show that an abnormal tTG-IgA drawn after initiation of a gluten-free diet is not correlated with duodenal mucosal injury in pediatric patients with CD and EGID. This suggests that serologic surveillance with tTG-IgA is not sufficient to monitor CD intestinal healing in this patient cohort. Persistent elevations of tTG-IgA in CD patients with normal duodenal biopsies should prompt investigation into other potential causes.

**What Is Known**

- Celiac disease (CD) patients have high levels of anti-tissue transglutaminase IgA (tTG-IgA) antibodies before diagnosis. These antibody levels decrease after the initiation of a gluten-free diet.
- CD causes histologic changes in the duodenum including intraepithelial lymphocytosis and villous blunting. These changes are reversible with a gluten-free diet.

**What Is New**

- For pediatric patients with CD and eosinophilic gastrointestinal disorders on a gluten-free diet, an abnormal tTG-IgA after CD diagnosis does not correlate with duodenal mucosal injury.
- Normal tTG-IgA level after starting a gluten-free diet in patients with established CD and eosinophilic gastrointestinal disorder does not ensure duodenal mucosal healing.
- Celiac serologic surveillance with tTG-IgA may not be sufficient to monitor CD status in this patient cohort.

## INTRODUCTION

Celiac disease (CD) is an immune-mediated enteropathy triggered by the presence of gluten in genetically susceptible individuals, with a prevalence in children as high as 1% (1–3). Currently, the only treatment for CD is a strict, lifelong gluten-free diet (GFD) (4). Diagnosis of CD in the United States is made through endoscopic proximal small-intestine biopsy to assess intraepithelial lymphocytosis, crypt hyperplasia, villous atrophy, and inflammation (1,5,6). While duodenal biopsy is the standard of care for CD diagnosis in North America, follow-up recommendations in pediatric patients with CD are limited (7). While current clinical guidelines do not recommend follow-up biopsies, there is no consensus on the value and role of repeat biopsy in the assessment of mucosal healing after beginning and maintaining a GFD for pediatric patients (5,8–10). Many pediatric practices rely solely on serologic findings such as tissue transglutaminase immunoglobulin A (tTG-IgA), and less commonly endomysial antibody IgA (EMA-IgA) and deamidated gliadin immunoglobulin G, following initial CD diagnosis in the assessment of duodenal healing. However, the reliability of serologies to assess degree of duodenal damage is unclear (1,2,4,11,12). Therefore, the accuracy of using tTG-IgA as a marker of duodenal mucosal healing and its potential correlation with symptoms, adherence to a GFD, and other factors require further investigation (13).

Current standard of care in the management of uncomplicated CD is not to undergo multiple esophagogastroduodenoscopies (EGDs). Therefore, it was important to identify a subsection of pediatric CD patients who have had multiple EGDs to investigate the relationship of biopsies and antibody levels over time. In this study, patients with both CD and eosinophilic gastrointestinal disorders (EGID) including eosinophilic esophagitis (EoE) and eosinophilic gastritis (EG) were identified to explore this relationship, as it is standard for patients with EGID to undergo repeat EGDs for disease surveillance (14,15).

In this study, data from pediatric patients with CD and EGID who have had multiple EGDs and duodenal biopsies are presented to assess duodenal healing before and after starting a GFD and correlate it with tTG-IgA serology. In this way, the reliability of using serologies to monitor improvement of duodenal damage can be assessed in this patient cohort.

## METHODS

A retrospective data analysis was conducted by obtaining patient data from the electronic medical record from the Children’s Hospital of Philadelphia (CHOP). According to the North American Society for Pediatric Gastroenterology, Hepatology, and Nutrition guidelines, a CD diagnosis is confirmed through characteristic pathology taken from the duodenal bulb and the distal duodenum (16). These microscopic features include infiltration of lymphocytes into the epithelium of the duodenum, progressive flattening of the villi, and deepening of the crypts (17). All patients included in this study met the North American Society for Pediatric Gastroenterology, Hepatology, and Nutrition criteria for CD and had positive levels of tTG-IgA at the time of diagnosis. All patients were started on a GFD after CD diagnosis. EGID consists of several EGID; the study patients all met criteria for either EoE or EG. EoE is an atopic disease characterized by eosinophils within the esophageal epithelium, leading to esophageal dysmotility and occasionally tissue fibrosis and stricture formation (18,19). EoE was defined by the presence of at least 15 eosinophils per high-powered field on esophageal biopsy either at the time of CD diagnosis or prior (14). EG was defined by the presence of eosinophils in the gastric mucosa (15).

### Patients

**FIGURE 1.**
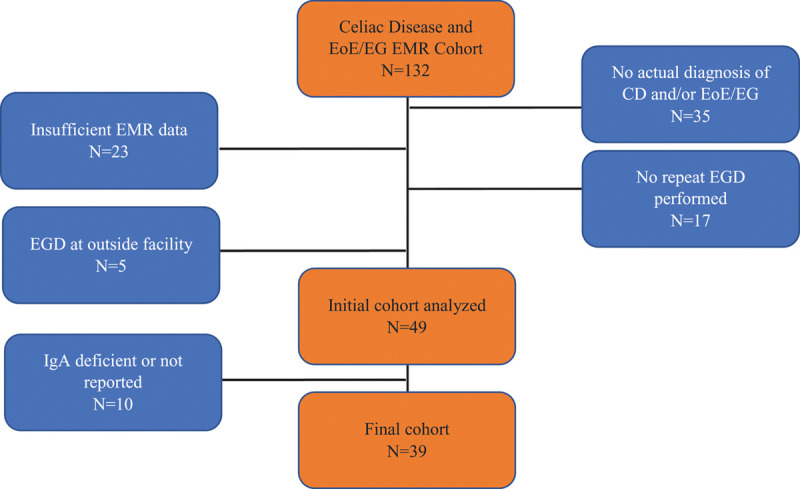
Flow chart of patient selection. CD = celiac disease; EG = eosinophilic gastritis; EGD = esophagogastroduodenoscopy; EMR = electronic medical record; EoE = eosinophilic esophagitis; IgA = immunoglobulin A.

Patients with International Classification of Diseases, Ninth Revision or International Classification of Diseases, Tenth Revision coded diagnoses of CD and EoE/EG seen at CHOP between 2003 and 2018 were included. Of these 132 patients, 35 did not meet the diagnostic criteria of CD or EoE/EG, as defined above. An additional 23 patients were eliminated due to insufficient data in the electronic medical record, 17 were excluded because they did not undergo a repeat EGD, 3 were excluded for not having repeat antibody levels, and 5 more were excluded for having EGD performed at outside hospitals. Additionally, 10 were eliminated for being IgA deficient or having no reported IgA level. The final analyzed cohort was 39 patients (Fig. 1). Eighty-two percent (32/39) of patients were diagnosed with CD and EGID at the same time.

### Celiac Serologies

Laboratories reviewed and analyzed from CHOP and other commercial laboratories included tTG-IgA, total IgA, and EMA-IgA. The average length of time between collection of laboratories and EGD was 30 days (median = 0 days). It should be noted that celiac serologies were analyzed at several different laboratories, each with unique tTG-IgA normal scales.

### Duodenal Biopsies

EGD pathology reports from diagnostic duodenal biopsies and repeat duodenal biopsies were reviewed and the presence of intraepithelial lymphocytes and degree of villous blunting was assessed. Villous blunting was classified as absent, mild, moderate, or severe based on pathologic report. The presence of increased intraepithelial lymphocytes was classified as either present or absent. Intraepithelial lymphocytes were considered to be increased if there were more than 40 lymphocytes per 100 enterocytes in the duodenum (17). Pathology reports did not consistently comment on crypt architecture/hyperplasia and this was therefore not analyzed.

### Clinical Manifestations

Clinic notes from office visits before initial EGD diagnostic of CD and before start of the GFD, as well as at time of second EGD and after initiation of the GFD, were reviewed. Clinical manifestations including abdominal pain, nausea, vomiting, diarrhea, constipation, flatus, gastroesophageal reflux, rashes, joint pain, and malnutrition were recorded. Malnutrition was defined as a *z* score of ≤−1.0 based on weight/height-for-age, body-mass-index-for-age, weight-for-height-for-age, or mid-upper-arm-circumference. Patients were considered malnourished if they had mild, moderate, or severe malnutrition.

### Statistical Analysis

The data were analyzed using the Fisher exact test and *t* test.

### Institutional Review Board Approval

This study was approved by the Institutional Review Board at CHOP.

## RESULTS

In total, 39 pediatric patients met the inclusion criteria of both CD and EGID and were IgA sufficient. All 39 had positive endoscopic findings indicative of CD and positive tTG-IgA at the time of diagnosis, and all 39 had repeat biopsies and serologies after beginning a GFD. As shown in Table 1, 92% (36/39) of patients were symptomatic at diagnosis, including 51% (20/39) with abdominal pain, and 38% (15/39) with malnutrition.

**TABLE 1.** Patient characteristics at diagnosis of CD (n = 39)

Characteristic	% (n)
Gender	
Female	31 (12)
Male	69 (27)
Mean age at diagnosis, y ± SD	7 ± 3
Type 1 diabetes	13 (5)
Asthma	15 (6)
Symptoms at diagnosis	92 (36)
Abdominal pain	51 (20)
Nausea and/or vomiting	13 (5)
Diarrhea	18 (7)
Constipation	31 (12)
Flatus	5 (2)
Gastroesophageal reflux	23 (9)
Rash	13 (5)
Joint pain	8 (3)
Malnutrition	38 (15)

CD = celiac disease; SD = standard deviation.

**FIGURE 2.**
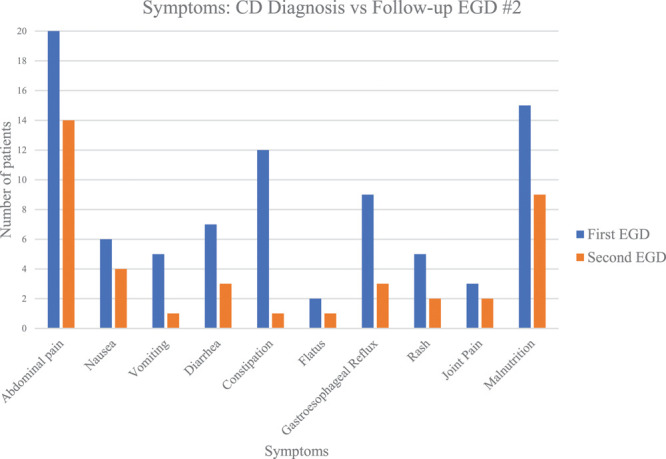
Frequency of symptoms at the time of initial CD diagnosis and second EGD after initiating a gluten-free diet. CD = celiac disease; EGD = esophagogastroduodenoscopy.

All study patients underwent at least 1 repeat EGD after CD diagnosis to assess EGID disease activity, as is standard of care for EGID. All patients had duodenal biopsies as well as esophageal biopsies during repeat EGD. At time of second EGD, 59% (23/39) reported ongoing symptoms and 33% (13/39) were asymptomatic. A symptom update was unavailable for 8% (3/39) of patients. Figure 2 depicts the patient-reported symptoms at time of first and second EGD. The average length of time between the first and second EGDs for the cohort was 324 days (the median was 175 days; the range was 31–1897 days). Fifty-six percent (22/39) of patients had a repeat endoscopy within 6 months. All symptoms improved for all patients between the first 2 EGDs. Abdominal pain, constipation, and malnutrition were the most frequently reported ailments at time of CD diagnosis. At time of second EGD, abdominal pain, nausea, and malnutrition were most commonly reported. Eighty-seven percent (34/39) were on a proton pump inhibitor at the time of second EGD, and 97% (38/39) were treated with a proton pump inhibitor at some point during the study timeframe. Fifty-nine percent (23/39) were treated with elimination diets for eosinophilic disease. Five percent (2/39) were treated with swallowed steroids, and 1 patient was treated with an inhaled steroid. Sixty-two percent (23/37) of patients still had evidence of active EoE at the time of second EGD (≥15 eosinophils per high powered field). Both patients with EG (2/2) still had evidence of eosinophilia in the stomach at the time of the second EGD.

**FIGURE 3.**
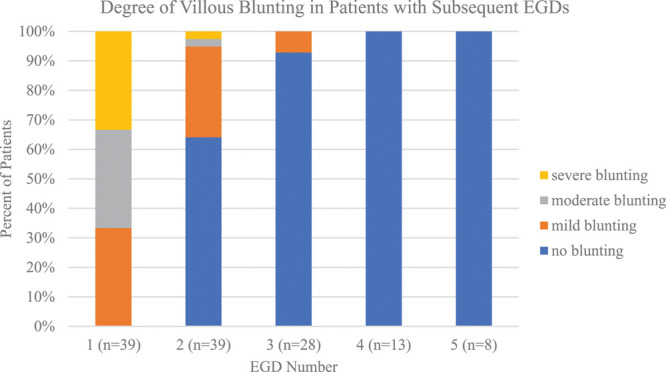
Relative proportions of patients with villous blunting on subsequent EGDs. EGD = esophagogastroduodenoscopy.

If available, data from up to 5 EGDs was collected for each patient. All 39 patients had at least 2 EGDs, 72% (28/39) had 3 EGDs, 33% (13/39) had 4 EGDs, and 21% (8/39) had 5 EGDs. Multiple EGDs were performed due to persistently active EGID. At the tie of second EGD, 36% (14/39) had mild to severe villous blunting; at the third EGD, 7% (2/28) had mild to severe villous blunting; and at the fourth and fifth EGD, no patients had any evidence of villous blunting on duodenal biopsy (Fig. 3). These data reinforce the principle that over time, duodenal architecture will return to normal in most patients with CD provided they maintain a strict GFD.

Forty-four percent of patients (17/39) had a normal second duodenal biopsy after CD diagnosis and starting a GFD (Table 2). Repeat biopsies were performed a mean of 324 days or 10.5 months after initial EGD. Fifteen of the 17 patients with normal repeat duodenal biopsies had tTG-IgA levels taken at the time of the second EGD, and of these 15 patients, 60% (9/15) had an abnormal tTG-IgA level. The relationship between tTG-IgA and duodenal pathology was completely independent in these 15 patients (*P* = 1.000). Of the nine patients with abnormal tTG-IgA and normal biopsies, 78% (7/9) had down-trending levels of tTG-IgA, 11% (1/9) had a higher tTG-IgA, and 11% (1/9) were not able to be compared due to subsequent bloodwork being drawn at different laboratories with unique reference ranges.

**TABLE 2.** Symptoms, serology, and histopathology results at first and second EGD

	N (%)	*P* value
First EGD		
Symptomatic at diagnosis	36/39 (92)	
Positive tTG-IgA	39/39 (100)	
Positive EMA-IgA	30/30 (100)	
Mild to severe duodenal villous blunting	39/39 (100)	
Second EGD		
Ongoing symptoms	23/39 (59)	
On PPI	34/39 (87)	
On an elimination diet	39/39 (100)	
Mild to severe duodenal villous blunting	14/39 (36)	
Active eosinophilic disease	23/39 (59)	
Normal repeat duodenal biopsy	17 /39 (44)	
Positive tTG-IgA	9/15* (60)	1.000†
Negative tTG-IgA	6/15* (40)	
Active CD on repeat duodenal biopsy	22/39 (56)	
Positive tTG-IgA	14/22 (64)	
Negative tTG-IgA	8/22 (36)	1.000†
Both increased intraepithelial lymphocytes and villous atrophy	8/39 (21)	
Positive tTG-IgA	6/8 (75)	0.324
Negative tTG-IgA	2/8 (25)	
Negative tTG-IgA	14/39 (36)	
Active CD on repeat duodenal biopsy	8/14 (57)	1.000
Positive tTG-IgA	21/39 (54)	
Normal repeat duodenal biopsy	9/21 (43)	1.000
Active EoE	4/9 (44)	
Gastritis	2/9 (22)	
No histologic pathology in esophagus, stomach or duodenum	3/9 (33)	
Negative EMA-IgA	17/32 (53)	
Abnormal repeat duodenal biopsy	2/17 (12)	0.106
Positive EMA-IgA	15/32 (47)	
Normal repeat duodenal biopsy	9/15 (60)	0.106

CD = celiac disease; EGD = esophagogastroduodenoscopy; EMA = endomysial antibody; EoE = eosinophilic esophagitis; PPI = proton pump inhibitor; IgA = immunoglobulin A; tTG = tissue transglutaminase.

*15 of 17 had tTG-IGA levels recorded.

†No correlation between tTG-IgA level and duodenal pathology.

Fifty-six percent (22/39) of patients had evidence of active CD with either villous atrophy or increased intraepithelial lymphocytes on second duodenal biopsy (Table 2). Of these, 21% (8/39) had both villous atrophy and intraepithelial lymphocytes. Thirty-six percent (8/22) of patients with active CD and 25% of patients with both pathologic findings (2/8) had normal tTG-IgA levels despite having duodenal mucosal injury on repeat biopsy (*P* = 1.000). These data indicate that there is no correlation between pathologic duodenal architecture and abnormal tTG-IGA antibody levels.

Forty-three percent (9/21) of patients with a positive tTG-IgA at the time of the second biopsy had a normal duodenal biopsy. Of these 9 patients with elevations of tTG-IgA, 44% (4/9) had active EoE, 22% (2/9) had gastritis, and the remaining 33% (3/9) had no evidence of active EoE, gastritis, or CD.

Thirty-six percent (14/39) of patients had negative tTG-IgA values at the time of their second biopsy. Of these, 57% (8/14) had evidence of active histologic duodenal disease at the time of second biopsy despite negative tTG-IgA levels.

In a subanalysis, patients whose repeat endoscopies were completed at least 1 year after CD diagnosis were evaluated. Sixty-seven percent (26/39) had a repeat biopsy taken at least 1 year after diagnosis and initiation of GFD, and 46% (18/39) had a repeat biopsy taken at least 2 years after diagnosis and initiation of GFD. Of these 18 patients, 94% (17/18) had normal duodenal biopsies after 2 years, and 83% (15/18) had normal tTG-IgA values after 2 years. Of note, the 1 patient with an abnormal biopsy had a normal tTG-IgA value, and the 3 patients with abnormal laboratory values had normal biopsies.

The reference values for tTG-IgA are not standardized. Rather, they vary between laboratories. In this patient cohort, 59% (23/39) had their initial bloodwork, and at least 1 repeat set of bloodwork, at CHOP’s laboratory which quantified “normal” range as 0–20 U/mL, and “abnormal” as above 20 U/mL. A subanalysis of these 23 patients was conducted to compare tTG-IgA levels over time after initiating a GFD. The average tTG-IgA value at diagnosis in this sub-group was 145.6 U/mL. Fifty-seven percent (13/23) had repeat laboratories drawn within 6 months of CD diagnosis (mean = 118 days). The average repeat tTG-IGA level within 6 months of starting a GFD was 42.3 U/mL. Sixty-five percent (15/23) of patients in this subgroup had repeat laboratories taken between 6 and 12 months from diagnosis (mean = 279 days). The average tTG-IgA level taken within 6–12 months of initiating a GFD was 26.8 U/mL. Forty-three percent (10/23) had repeat laboratories taken 12 months or more after diagnosis (mean = 826 days). The average tTG-IgA value of these patients was 22.8 U/mL.

At the time of the first EGD, 30 of 39 patients had an EMA-IgA collected. All 30 patients had a positive EMA-IgA. At the time of the second EGD, 32 of 39 total patients had an EMA-IgA collected. Forty-seven percent (15/32) had a positive EMA-IgA. Sixty percent (9/15) had a positive EMA-IgA and normal duodenal pathology on the second EGD. Twelve percent (2/17) had a negative EMA value at the time of the second EGD and also had an abnormal duodenal biopsy (*P* = 0.106).

Twenty-three percent (9/39) of patients in the study never had a normal biopsy indicating CD remission. Of these 9 patients, 5 became symptom-free on a GFD, and therefore did not undergo additional endoscopies. Three patients were lost to follow-up. The final patient had a follow-up EGD but the physician did not take duodenal biopsies. Rather, the physician noted that the duodenum was visually normal, compared with previously abnormal visual findings. All patients that had 4 or 5 EGDs achieved histologic remission. The average length of time from initial abnormal biopsy and CD diagnosis to duodenal healing in those that did achieve duodenal healing was a mean of 565 days and a median of 297 days (the mean is skewed due to outliers with prolonged healing times). Eight percent (3/39) of patients had a normal duodenal biopsy, but later had an abnormal biopsy during repeat EGD for persistent EGID.

## DISCUSSION

CD is a life-long immune-mediated enteropathy with the potential to lead to many chronic health complications without proper management. While the current standard of care in pediatric gastroenterology is to monitor serologic markers following diagnosis to predict gastrointestinal healing, there is conflicting evidence as to whether serology appropriately reflects the degree of duodenal damage, and therefore whether a child’s CD is being adequately controlled. Current guidelines from the American Gastroenterological Society recognize the potential unreliability of tTG-IgA, and recommend repeat biopsy in patients with persistent or relapsing symptoms even with negative serology (20).

Long-term consequences of uncontrolled CD, including nutritional deficiencies, increased risk of lymphoma, low bone density, dermatologic rashes, and ongoing gastrointestinal symptoms, indicate that systematic follow-up and monitoring are important in the management of pediatric CD (4,11,21,22). Data collected in the United States regarding mucosal healing are highly variable and suggest that serology levels may not be reliable markers of pathology (1,4,10). Since the standard of care for patients diagnosed with CD alone does not currently include repeat EGD, evaluating pathology data for patients diagnosed with both CD and EGID provides biopsy data on CD patients before and after initiation of a GFD.

This pilot study demonstrated that in patients with pediatric CD and EGID, abnormal tTG-IgA and EMA-IgA antibody levels do not correlate with abnormal duodenal pathology after initiation of a GFD. If a duodenal biopsy was found to be normal on the second EGD, only 40% of the patients had a normal tTG-IgA and 60% had a normal EMA-IgA. Some pediatric patients still demonstrated characteristic changes on duodenal biopsy at the time of repeat EGD, such as increased intraepithelial lymphocytes and villous blunting, and yet had normal tTG-IgA and EMA-IgA values. Thus, for pediatric patients with CD and EGID, abnormal celiac serologies after initiating a GFD may not correlate with duodenal mucosal injury and may not be a reliable predictor of duodenal healing. Therefore, healthcare providers should be cautious of relying solely on celiac serologies, particularly tTG-IgA, as markers to assess duodenal mucosal healing in children with CD. Further consideration should be made regarding repeat duodenal biopsies to confirm mucosal healing.

The vast majority of patients who had biopsies taken at least 2 years after their initial diagnosis of CD had tTG-IgA levels and duodenal structure that normalized. The normalization of previously damaged duodenal mucosa in this study reinforces the principle that small bowel inflammation is reversible for children with CD who maintain a strict lifelong GFD (23,24). Furthermore, these findings reinforce that clinicians should temper expectations of mucosal healing and serologic normalization in children with CD before 2 years on a GFD.

Questions arise as to what could be driving elevated tTG-IgA in patients who no longer have evidence of active CD on duodenal biopsies. Of the patients with elevated tTG-IgA and normal duodenal biopsies, 66% (6/9) had inflammation elsewhere in the upper gastrointestinal tract, including 4 patients with active EOE and 2 patients with gastritis. This raises the question as to whether EoE or other inflammatory conditions of the upper gastrointestinal tract could also elevate tTG-IgA in CD patients, even after their CD has gone into remission. Several studies have investigated and hypothesized potential causes of elevated tTG-IgA in pediatric patients without CD, including increased polyclonal IgA production in children with juvenile idiopathic arthritis, an acute immunologic phenomenon in response to infectious disease, and in response to fibrogenesis and apoptosis in patients with end-stage heart failure (25–28). Additionally, recent studies have reported elevated serum tTG-IgA in non-CD patients with EoE (29). The findings of this study support the aforementioned literature. Further investigation into the relationship of tTG-IgA and eosinophilic disorders of the gastrointestinal tract is warranted.

tTG-IgA remains an important laboratory test to screen for CD. High levels of tTG-IgA in a patient who has not yet been diagnosed with CD should warrant further workup with an EGD. CD serologic surveillance with tTG-IgA, with or without EMA-IgA, may not be sufficient to monitor CD status in children with CD and EGID, and further consideration should be made regarding repeat duodenal biopsies to assess mucosal healing.

Limitations of this study include its retrospective nature, relatively small cohort size, and potential confounding variable of concomitant EGID diagnosis. Additionally, tTG-IgA levels were analyzed at several different laboratories, each with unique normal range scales. The study participants were primarily male, possibly due to EoE being more common in males, which is a possible confounding factor (30). Given the data from this retrospective study, additional prospective research is needed to further validate these findings.

## ACKNOWLEDGMENTS

We acknowledge and thank Lionola Juste for her efforts in reviewing the manuscript before submission. K.P. contributed to the substantial contributing to conception or design of the work, data analysis, drafting manuscript, revision of manuscript, final approval of published version, agreed to be accountable for all aspects of work and approved the final draft. L.R. contributed to data analysis, drafting manuscript, revision of manuscript, final approval of published version, agreed to be accountable for all aspects of work and approved the final draft. P.B. contributed to the substantial contributing to conception or design of the work, revision of manuscript, final approval of published version, agree to be accountable for all aspects of work and approved the final draft. K.F. contributed to the substantial contributing to conception or design of the work, revision of manuscript, final approval of published version, agreed to be accountable for all aspects of work and approved the final draft. J.S. contributed to the substantial contributing to conception or design of the work, revision of manuscript, final approval of published version, agreed to be accountable for all aspects of work and approved the final draft. K.H. contributed to the substantial contributing to conception or design of the work, revision of manuscript, final approval of published version, agreed to be accountable for all aspects of work and approved the final draft. A.F. contributed to the substantial contributing to conception or design of the work, revision of manuscript, final approval of published version, agreed to be accountable for all aspects of work and approved the final draft. R.V. contributed to the substantial contributing to conception or design of the work, revision of manuscript, final approval of published version, agreed to be accountable for all aspects of work and approved the final draft. A.S. contributed to the substantial contributing to conception or design of the work, revision of manuscript, final approval of published version, agreed to be accountable for all aspects of work and approved the final draft. L.M.F. contributed to the substantial contributing to conception or design of the work, data analysis, drafting manuscript, revision of manuscript, final approval of published version, agreed to be accountable for all aspects of work and approved the final draft.

## References

[1] Bannister EG Cameron DJ Ng J. Can celiac serology alone be used as a marker of duodenal mucosal recovery in children with celiac disease on a gluten-free diet? Am J Gastroenterol. 2014; 109:1478–1483. 25070050 10.1038/ajg.2014.200

[2] Elitsur Y Sigman T Watkins R. Tissue transglutaminase levels are not sufficient to diagnose celiac disease in North American practices without intestinal biopsies. Dig Dis Sci. 2017; 62:175–179. 27778203 10.1007/s10620-016-4354-4

[3] Vaquero L Rodríguez-Martín L Alvarez-Cuenllas B. Coeliac disease and gastrointestinal symptom screening in adult first-degree relatives. J Gastroenterol Hepatol. 2017; 32:1931–1937. 28387454 10.1111/jgh.13801

[4] Bai JC Fried M Corazza GR; World Gastroenterology Organization. World Gastroenterology Organisation global guidelines on celiac disease. J Clin Gastroenterol. 2013; 47:121–126. 23314668 10.1097/MCG.0b013e31827a6f83

[5] Bozzola M Meazza C Gertosio C. Omitting duodenal biopsy in children with suspected celiac disease and extra-intestinal symptoms. Ital J Pediatr. 2017; 43:59. 28709446 10.1186/s13052-017-0377-5 PMC5512979

[6] Ermarth A Bryce M Woodward S. Identification of pediatric patients with celiac disease based on serology and a classification and regression tree analysis. Clin Gastroenterol Hepatol. 2017; 15:396–402.e2. 27847281 10.1016/j.cgh.2016.10.035 PMC5316297

[7] Snyder J Butzner JD DeFelice AR. Evidence-informed expert recommendations for the management of celiac disease in children. Pediatrics. 2016; 138:e20153147. 27565547 10.1542/peds.2015-3147

[8] Leonard MM Weir DC DeGroote M. Value of IgA tTG in predicting mucosal recovery in children with celiac disease on a gluten-free diet. J Pediatr Gastroenterol Nutr. 2017; 64:286–291. 28112686 10.1097/MPG.0000000000001460 PMC5457911

[9] Rubio-Tapia A Rahim MW See JA. Mucosal recovery and mortality in adults with celiac disease after treatment with a gluten-free diet. Am J Gastroenterol. 2010; 105:1412–1420. 20145607 10.1038/ajg.2010.10 PMC2881171

[10] Wolf J Petroff D Richter T. Validation of antibody-based strategies for diagnosis of pediatric celiac disease without biopsy. Gastroenterology. 2017; 153:410–419.e17. 28461188 10.1053/j.gastro.2017.04.023

[11] Hill ID Dirks MH Liptak GS. North American Society for Pediatric Gastroenterology, Hepatology and Nutrition. Guideline for the diagnosis and treatment of celiac disease in children: recommendations of the North American Society for Pediatric Gastroenterology, Hepatology and Nutrition. J Pediatr Gastroenterol Nutr. 2005; 40:1–19. 15625418 10.1097/00005176-200501000-00001

[12] Silvester JA Kurada S Szwajcer A. Tests for serum transglutaminase and endomysial antibodies do not detect most patients with celiac disease and persistent villous atrophy on gluten-free diets: a meta-analysis. Gastroenterology. 2017; 153:689–701.e1. 28545781 10.1053/j.gastro.2017.05.015 PMC5738024

[13] Ghazzawi Y Rubio-Tapia A Murray JA. Mucosal healing in children with treated celiac disease. J Pediatr Gastroenterol Nutr. 2014; 59:229–231. 24691402 10.1097/MPG.0000000000000390

[14] Merves J Muir A Modayur Chandramouleeswaran P. Eosinophilic esophagitis. Ann Allergy Asthma Immunol. 2014; 112:397–403. 24566295 10.1016/j.anai.2014.01.023 PMC4332835

[15] Prussin C. Eosinophilic gastroenteritis and related eosinophilic disorders. Gastroenterol Clin North Am. 2014; 43:317–327. 24813518 10.1016/j.gtc.2014.02.013 PMC4130565

[16] Hill ID Fasano A Guandalini S. NASPGHAN clinical report on the diagnosis and treatment of gluten-related disorders. J Pediatr Gastroenterol Nutr. 2016; 63:156–165. 27035374 10.1097/MPG.0000000000001216

[17] Hujoel IA Reilly NR Rubio-Tapia A. Celiac disease: clinical features and diagnosis. Gastroenterol Clin North Am. 2019; 48:19–37. 30711209 10.1016/j.gtc.2018.09.001

[18] Fahey L Guzek R Ruffner MA. EMSY gene silencing in the esophageal epithelium. Gastroenterology. 2017; 152:S855.

[19] Rothenberg ME Spergel JM Sherrill JD. Common variants at 5q22 associate with pediatric eosinophilic esophagitis. Nat Genet. 2010; 42:289–291. 20208534 10.1038/ng.547 PMC3740732

[20] Husby S Murray JA Katzka DA. AGA clinical practice update on diagnosis and monitoring of celiac disease-changing utility of serology and histologic measures: expert review. Gastroenterology. 2019; 156:885–889. 30578783 10.1053/j.gastro.2018.12.010 PMC6409202

[21] Belei O Dobrescu A Heredea R. Histologic recovery among children with celiac disease on a gluten-free diet. A long-term follow-up single-center experience. Arch Med Sci. 2018; 14:94–100. 29379538 10.5114/aoms.2018.72241 PMC5778430

[22] Jansen M van Zelm M Groeneweg M. The identification of celiac disease in asymptomatic children: the Generation R Study. J Gastroenterol. 2018; 53:377–386. 28589338 10.1007/s00535-017-1354-x PMC5847176

[23] Szakács Z Mátrai P Hegyi P. Younger age at diagnosis predisposes to mucosal recovery in celiac disease on a gluten-free diet: a meta-analysis. PLoS One. 2017; 12:e0187526. 29095937 10.1371/journal.pone.0187526 PMC5695627

[24] Hære P Høie O Schulz T. Long-term mucosal recovery and healing in celiac disease is the rule - not the exception. Scand J Gastroenterol. 2016; 51:1439–1446. 27534885 10.1080/00365521.2016.1218540

[25] Stoll ML Patel AS Christadoss ML. IgA transglutaminase levels in children with Juvenile Idiopathic Arthritis. Ann Paediatr Rheumatol. 2012; 1:31–35. 23750324 10.5455/apr.112220111551 PMC3675655

[26] Ferrara F Quaglia S Caputo I. Anti-transglutaminase antibodies in non-coeliac children suffering from infectious diseases. Clin Exp Immunol. 2010; 159:217–223. 19912255 10.1111/j.1365-2249.2009.04054.x PMC2810390

[27] Peracchi M Trovato C Longhi M. Tissue transglutaminase antibodies in patients with end-stage heart failure. Am J Gastroenterol. 2002; 97:2850–2854. 12425559 10.1111/j.1572-0241.2002.07033.x

[28] Tola MD Parillà F Trappolini M. Antitissue transglutaminase antibodies in acute coronary syndrome: an alert signal of myocardial tissue lesion? J Intern Med. 2007; 263:43–51. 10.1111/j.1365-2796.2007.01881.x 18088251

[29] Le Fevre AK Walker MM Hadjiashrafy A. Elevated serum tissue transglutaminase antibodies in children with eosinophilic esophagitis. J Pediatr Gastroenterol Nutr. 2017; 65:69–74. 28644352 10.1097/MPG.0000000000001437

[30] Dellon ES. Epidemiology of eosinophilic esophagitis. Gastroenterol Clin North Am. 2014; 43:201–218. 24813510 10.1016/j.gtc.2014.02.002 PMC4019938

